# Surgical treatment of a rare bilateral synovial chondromatosis

**DOI:** 10.4322/acr.2020.183

**Published:** 2020-09-02

**Authors:** Matheus Dantas de Araújo Barretto, Shajadi Carlos Pardo Kaba, Fernando Melhem Elias, Maria Cristina Zindel Deboni

**Affiliations:** a Universidade de São Paulo (USP), Faculdade de Odontologia, Departamento de Cirurgia Prótese e Traumatologia Maxilofaciais. São Paulo, SP, Brazil; b Universidade de São Paulo (USP), Hospital Universitário, Divisão de Odontologia. São Paulo, SP, Brazil

**Keywords:** Chondromatosis, Synovial, Osteochondromatosis, Pathology, Oral, Temporomandibular Joint, Temporomandibular Joint Disorders, Review

## Abstract

Synovial chondromatosis (SC) in the temporomandibular joint (TMJ) is an uncommon entity, mostly when the involvement is bilateral. The authors report a rare case of bilateral SC, with a follow-up of 13 months, and a literature review. A 60-year-old Caucasian woman, with the chief complaint of pain for 6 years in the bilateral pre-auricular region, had a progressive clacking and discomfort on the left side during mouth opening. The panoramic image was suggestive of SC. The bilateral lesion was surgically removed by direct access. Histopathological examination confirmed the clinical diagnosis of bilateral SC. This article shows the importance of a multidisciplinary approach for the early diagnosis and appropriate treatment. Also, it encourages the referral of such cases to professionals with a greater familiarity with this entity.

## INTRODUCTION

Synovial chondromatosis (SC) is a cartilaginous metaplasia of mesenchymal remnants present in the articular synovial tissue.^1^ The main characteristic is the development of cartilaginous nodules in the synovial membrane.^1^ ^-^ ^3^ It typically affects large joints and only rarely involves the temporomandibular joint (TMJ).^1^ The bilateral involvement of the TMJ is even rarer.

The exact etiology of SC is unknown. However, in many reported cases, there is an association with other joint conditions including (i) degenerative arthritis, (ii)inflammatory joint disease, (iii) noninflammatory arthropathy, (iv) embryologic disorder, (v) infection, and (vi) trauma (secondary SC). When no etiological factors are identified, the cases are classified as primary SC.^1^ ^,^ ^2^

Clinically, SC is generally unilateral, more prevalent in women, and the right TMJ is mostly affected.^1^ ^-^ ^3^ SC affects mostly middle-aged individuals, although a wide age range was reported. Pain, swelling of the preauricular region, limitation of mouth opening, and crepitation are the common symptoms.^1^ ^-^ ^3^

Diagnostic imaging methods such as computed tomography and magnetic resonance imaging are important for diagnosis.^1^ ^-^ ^7^ However, definitive diagnosis is made through clinical correlation with imaging and histopathological examination.^1^ ^,^ ^7^

Due to the paucity of bilateral cases reported in the literature, we report a rare case of bilateral SC with 13 months of follow-up after treatment and a literature discussion.

## CASE REPORT

A 60-year-old Caucasian woman with the chief complaint of pain for 6 years in the bilateral pre-auricular region was referred to the Maxillofacial Surgery Department of the University of São Paulo Hospital. She reported that she visited several dentists, who recommended care in mouth opening and feeding, without any improvement. Additionally, another clinician prescribed pain killers (acetaminophen, ibuprofen, and tramadol) six months prior to our appointment. However, her symptoms remained unchanged. Past medical history included hypertension, depression, and referred osteoarthritis of the right knee, and was regularly taking 25 mg atenolol and 100 mg sertraline, daily. The patient denied a history of trauma or parafunctional habits.

**Figure 1:**
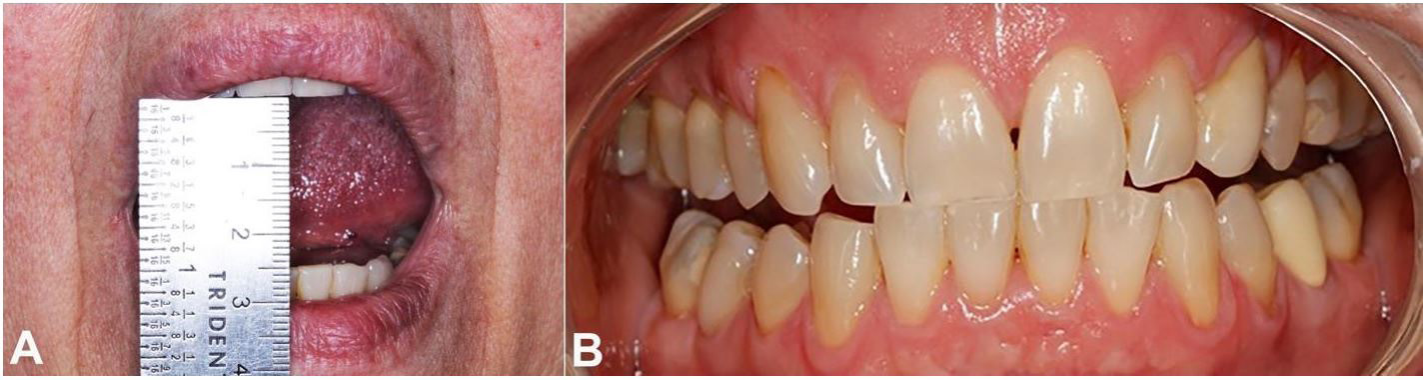
**A** – Oral examination - Mouth opening limitation; **B** – Posterior open-bite.

**Figure 2:**
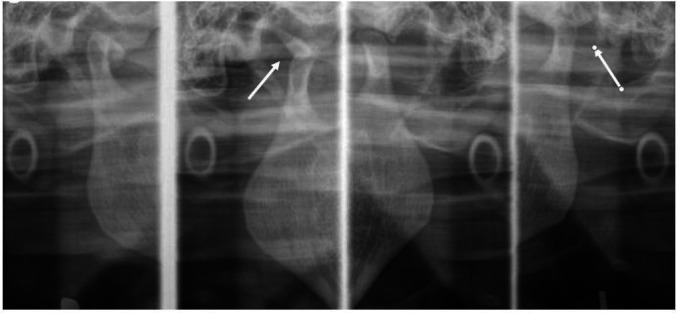
Panoramic X-ray showing Loose bodies on the right and left side of TMJ (arrows).

**Figure 3:**
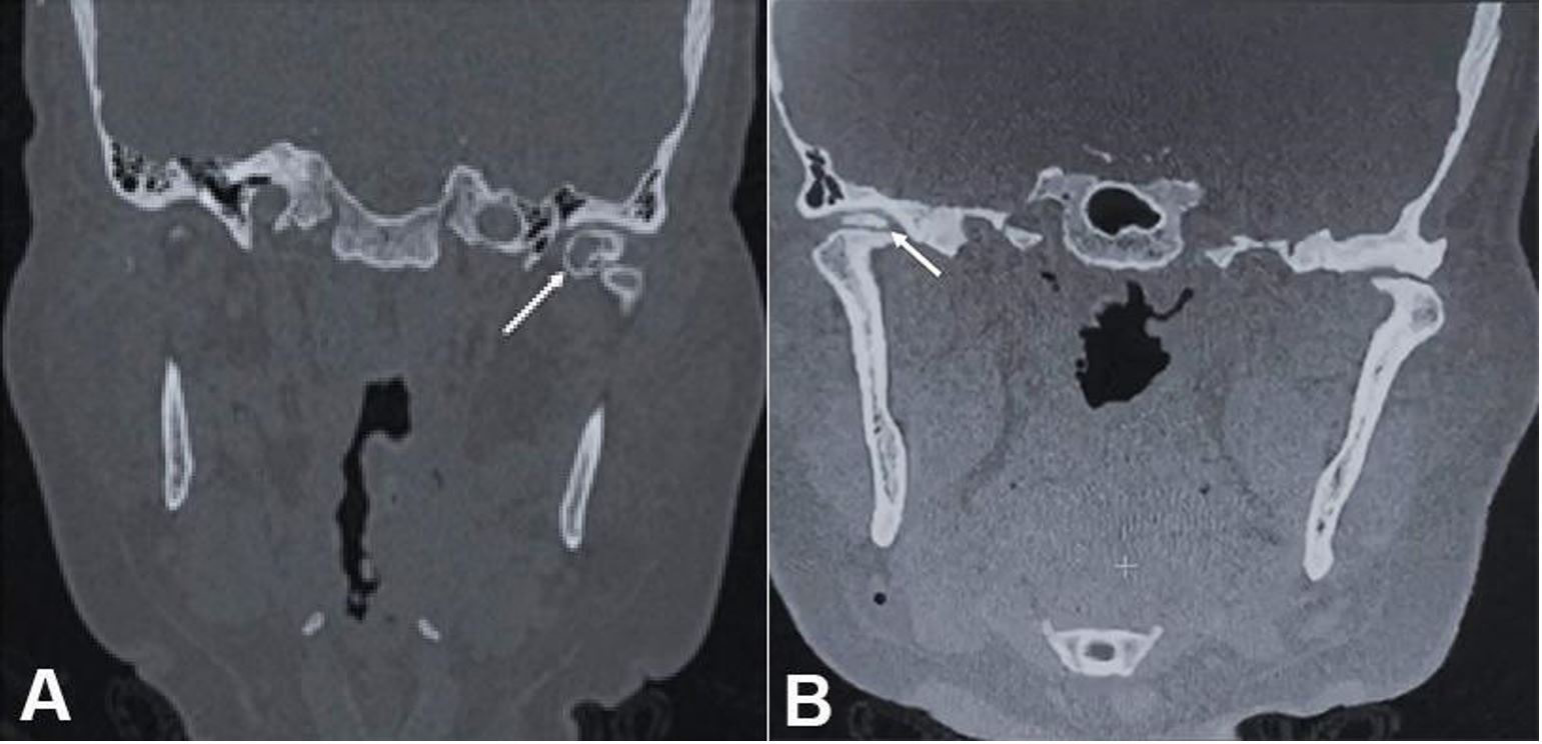
**A** – Preoperative computed tomography coronal section TMJ left side showing loose body (arrow); **B** – Preoperative computed tomography coronal section TMJ right side showing loose body (arrow).

Clinical examination revealed a limited mouth opening of 25 mm (Figure 1A), clicking in the right TMJ, and intense bilateral articular pain. Some teeth were absent, there was occlusal contact only at anterior teeth, and the median line was aligned (Figure 1B). TMJ radiography revealed radiopaque images in both TMJs (Figure 2). The CT confirmed the presence of loose bodies in TMJ bilaterally. Those bodies measured approximately 13 mm in the left, and 8mm in the right side (Figure 3A and 3B). There was a reduction of TMJ space associated with sclerosis. Due to the slow evolution process associated with clinical and imaging characteristics, the diagnostic hypothesis of bilateral SC was raised.

**Figure 4:**
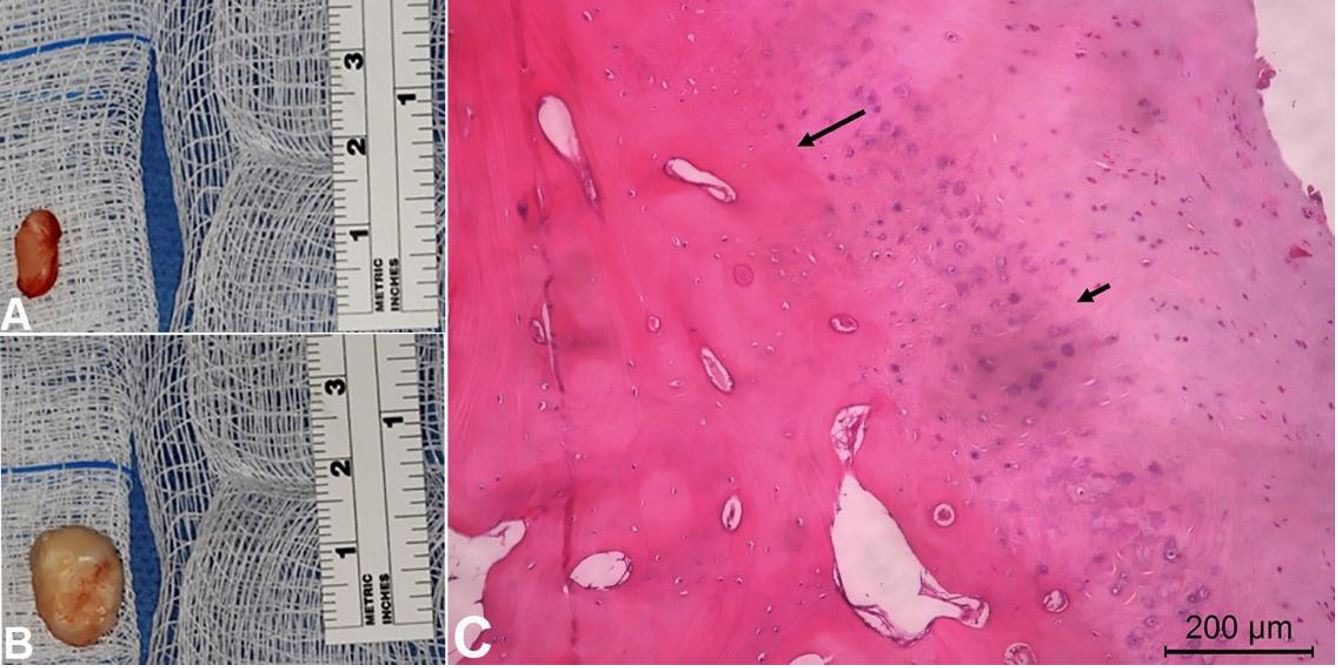
**A** and **B** – gross view of the extracted loose bodies; **C** – Photomicrograph of the loose body exhibiting hyaline cartilaginous formation (small arrow) and transitional zone to bone tissue characterizing a chondrometaplasia (arrow) (H&E, 400X).

Under general anesthesia, the TMJs were accessed directly by pre-auricular incision with bilaterally endaural extension, allowing for a good trans-surgical visual approach and good postoperative healing. Cartilaginous bodies of each joint were removed and analyzed (Figure 4A and 4B). They were characterized by irregular surface and stony consistency. There was no evidence of alteration of the condyles or the fossa. Histopathological examination revealed fragments of lamellar bone tissue in continuity with fibrocartilaginous tissue, surrounded by fibrous connective tissue (Figure 4C), confirming the diagnosis of bilateral SC.

**Figure 5:**
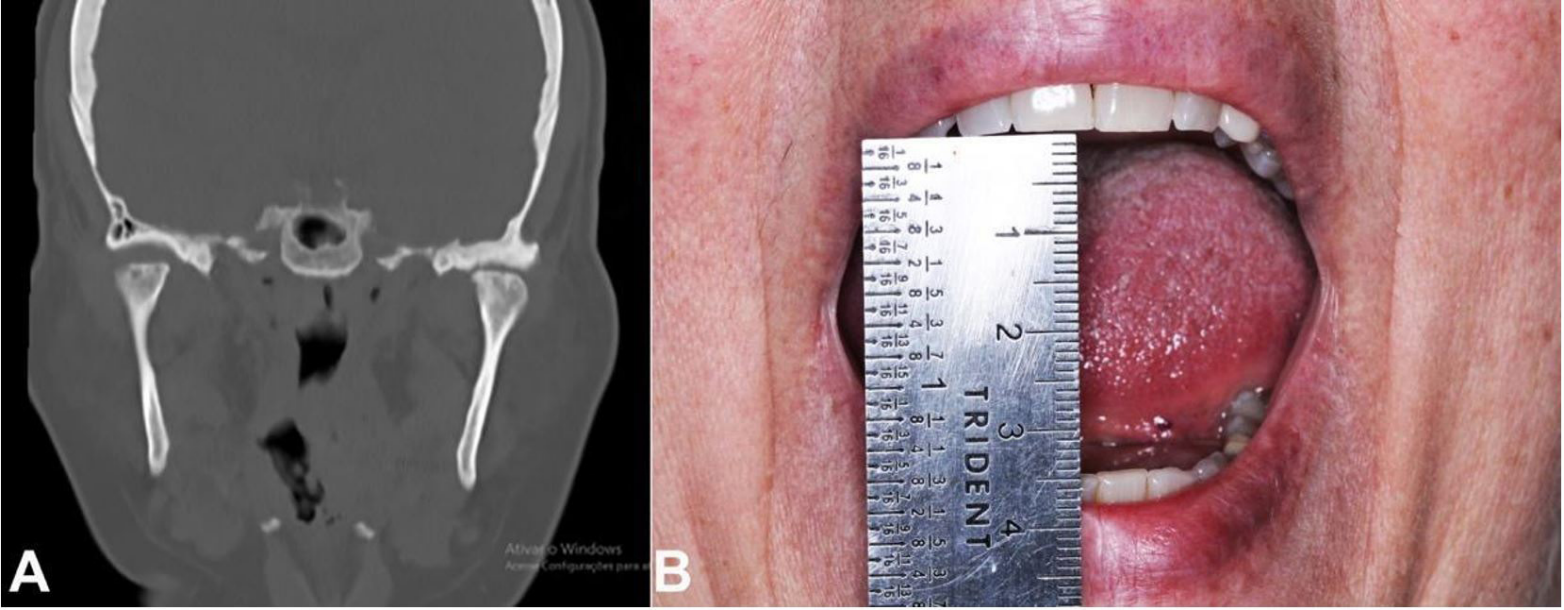
**A** – 13-months post-operative CT image showing the absence of loose bodies; **B** – Mouth opening improvement after 13-months post-operatory.

**Figure 6:**
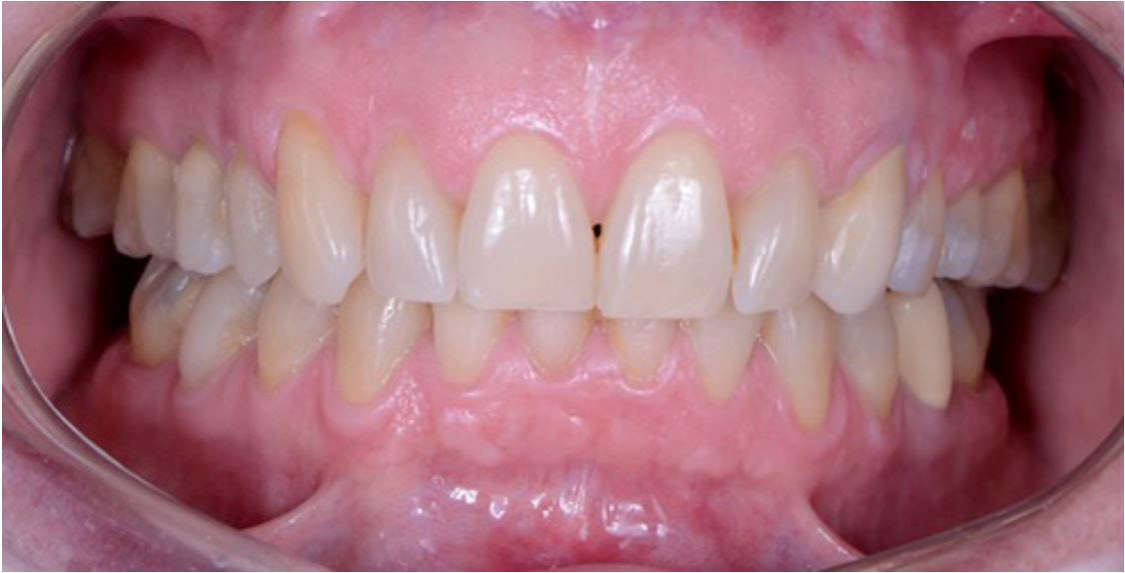
Oral examination showing the occlusion after 13-months after the surgical procedure.

CT performed after 13 months of surgery showed both joints with the absence of loose bodies (Figure 5A). The patient experienced improvement of mouth opening at 35 mm (Figure 5B), as well as the preservation of protrusive and latero-protrusive movements. Occlusion was reestablished (Figure 6). She remains without articular clicks, pain, or facial nerve deficit, with no signs of recurrence.

## DISCUSSION

A comprehensive review of SC in the TMJ was published in 2011 by Shah et al.,^1^ who studied 242 cases over 246 years. Since then, two new cases of bilateral SC were found in a systematic search in the PubMed and SCOPUS databases with the uniterms “(synovial chondromatosis) AND TMJ”.^2^ ^,^ ^4^ As far as we could get, including our case, there are only 5 cases of bilateral SC.

The etiology of SC is still a matter of discussion. Facial trauma, chronic renal disease, and arthrosis have been associated with SC.^1^ ^,^ ^3^ Similar to other TMJ entities, SC seems to be a multifactorial disease, induced by a marked matrix histochemical alteration, biochemical composition, and/or cellular metabolism.^8^ The molecular background is still uncertain, but the disease is thought to originate from a metaplastic variation of synovial cells into a cartilaginous tissue.^9^ A cytogenic study of large joints with SC revealed consistent abnormalities within chromosome 6, that suggested a neoplastic origin.^9^ However, no genetic correlation has been made in current literature related to TMJ SC.

The increased presence of growth factors such as fibroblast growth factor 2, 3, and 9; and receptors 3 and 1 have been mentioned in previous studies,^5^ which seem to promote the lesion growth. Moreover, bone morphogenetic proteins 2 and 4 were shown in cartilaginous nodules suggesting that bone morphogenetic proteins synthetized by the synovia and by the loose bodies promote bone and cartilaginous metaplasia. This contributes to the pathogenesis of SC.^3^ ^,^ ^5^ Similar to chondrosarcoma and chondroma of soft tissues, SC belongs to the group of extra-skeletal chondrogenic neoplasms.^1^ ^,^ ^3^ Hyaline cartilage is absent on the joint surface of the normal TMJ. Therefore, any presence of cartilaginous tissue favors metaplasia.^3^

Our patient had a history of osteoarthritis. This would classify this case as secondary SC, which is not a common association considering previously reported cases of TMJ SC.^1^ ^,^ ^2^ In studies with large joints, some researchers showed an association between a systemic entity, such as osteoarthritis and SC.^7^ Bilateral involvement is extremely rare, and its etiology is believed to be the same as in the unilateral cases.^2^ ^-^ ^4^

Our case followed the epidemiological profile of SC. Women are most affected, in a ratio of 2:1.^1^ ^,^ ^3^ The 4^th^ and 5^th^ decades of life remain the most frequently associated with the disease.^1^ ^,^ ^3^ Higher prevalence in women in this age group may be associated with hormonal changes observed at menopause. As observed in studies with larger joints, some showed an association between systemic conditions and SC.^7^ This relationship is also suggested in the current study, but requires further investigation.

The symptoms of SC named as the “triad of disease” are pain, mouth opening limitation, and preauricular swelling on the affected side.^1^ ^,^ ^3^ The patient, in this study, presented with pain, limitation of mouth opening, crepitation, and posterior open bite. Lateral deviation during mouth opening was also reported. The non-specificity of symptoms and the rarity of the disease ultimately led to late diagnosis. Patients are usually over prescribed with symptomatic medication, without relief.

Diagnosis should include clinical examination, panoramic images, computerized tomography and/or magnetic resonance images.^4^ Arthroscopy is also an excellent diagnostic tool.^2^ Definitive diagnosis is made through clinical correlation with imaging and histopathological exams.

Initially, we observed radiopaque images in both temporomandibular joints on the panoramic radiograph. Depending on the grade of calcification and size, loose bodies cannot be easily seen in CT images. We found loose bodies reducing synovial space. High-quality MRI images^6^ are valuable when loose bodies are smaller than 1 mm. In addition, sectional images are important in planning a surgical approach.

The treatment of choice for SC is the surgical excision,^1^ ^,^ ^3^ and direct extra-oral access is the most applied approach .^1^ ^,^ ^3^ Although some authors advocate the use of arthroscopy,^3^ ^,^ ^5^ arguing that it is less invasive, there is a possible limitation of trans-surgical visualization and incomplete removal of cartilaginous bodies.^1^ Direct surgical access allows better visualization for excision and less possibility of relapse.^1^ ^,^ ^7^

The main objective of surgery is the removal of loose bodies and the preservation of the joint structure. In exceptional cases, local synovectomy may be sufficient after accurate trans-surgical localization of the metaplastic area of the synovial membrane, in the early stage of chondromatosis. An aggressive approach, with condylectomy, is rarely indicated. Morphological changes of the condyle, associated with severe functional limitation, indicate arthroplasty.^2^ Intracranial extension has been observed in some chronic non-treated cases; therefore, it is imperative to periodically follow-up the patients.^1^ ^,^ ^3^ We recommend an extended follow-up after arthroscopy or a direct surgical approach when the surgeon is not certain that the lesion was accessed and removed.

Therefore, surgical treatment is the ideal treatment option for SC, with functional restoration of the patients. Adjuvant treatments such as physiotherapy and laser therapy to restore the patient’s joint function may be necessary in cases where there is a limitation of the mouth opening, limitation of protrusive and latero-protrusive movements, and occlusal alteration after the surgical treatment.^10^ In this case, the patient was advised to engage in physiotherapy exercises to practice opening and closing the mouth.

## CONCLUSIONS

Due to the scarcity of cases, it is important for clinicians to report cases of SC. As early symptoms are mild and can resemble many other diseases, the correct diagnosis and treatment are often delayed, resulting in disabling consequences for the patient. This creates discomfort and functional disabilities, besides the possibility of erosion of the cranial base.

All procedures performed in studies involving human participants were by the ethical standards of the institutional and/or national research committee and with the 1964 Helsinki declaration and its later amendments or comparable ethical standards.
